# Modeling and Control of a Two-Axis Stabilized Gimbal Based on Kane Method

**DOI:** 10.3390/s24113615

**Published:** 2024-06-03

**Authors:** Qixuan Huang, Jiaxing Zhou, Xiang Chen, Youxin Yao, Yuhao Chen, Wei Chen, Runjing Chen, Zhisheng Lv

**Affiliations:** 1 School of Electrical Engineering and Automation, Xiamen University of Technology, Xiamen 361024, China; 2222031375@stu.xmut.edu.cn (Q.H.); 2222031352@stu.xmut.edu.cn (Y.Y.); 2322131010@stu.xmut.edu.cn (Y.C.); 2322131007@stu.xmut.edu.cn (W.C.); 2014110602@xmut.edu.cn (Z.L.); 2 Xiamen Key Laboratory of Frontier Electric Power Equipment and Intelligent Control, Xiamen 361024, China; 3 Shanghai Institute of Satellite Engineering, Shanghai 201109, China; 2152.050350@163.com; 4 School of Computer and Information Engineering, Xiamen University of Technology, Xiamen 361024, China; chenrj@xmut.edu.cn

**Keywords:** two-axis stable system, Kane method, NPSO-PID

## Abstract

A two-axis stabilizing gimbal is a device that ensures a sensor is working properly on a moving platform. When classical mechanics (Newton–Euler and Lagrange) is employed to model a two-axis stable gimbal, its limitations can complicate the modeling process. To address this issue, a method for establishing a dynamic model for a two-axis stabilizing platform based on the Kane method is proposed in this paper. The Kane method offers the advantage of a simple model structure and computational efficiency. Initially, utilizing a generalized coordinate system, expressions of the generalized velocities, deflection velocities and angular velocities are derived. Subsequently, the generalized active forces and inertial forces acting on the two-axis stabilized gimbal are analyzed. Finally, by combining force and velocity with the Kane equation, the dynamic model of the two-axis stable platform is obtained, demonstrating the validity of the Kane method for establishing the two-axis stable platform model. To ensure the pointing accuracy stability of the two-axis stabilizing platform, a Novel Particle Swarm Optimization Proportion Integration Differentiation (NPSO-PID) controller is designed using the PSO algorithm. It is then simulated in MATLAB/Simulink and compared with a classical PID controller. Simulation results demonstrate that NPSO-PID exhibits superior object tracking performance compared to classical PID controllers and better optimization of control parameters compared to traditional PSO-PID controllers.

## 1. Introduction

The inertial stabilization of sensors is important in many applications, such as guided missiles, navigation systems [1], and tracking systems [2]. With the development and achievements in science and technology, more and more sensors are needed to work on mobile carriers. However, the movement of these carriers can interfere with the sensors’ measurement accuracy. In such an environment, where the sensors are mounted on a movable platform, maintaining sensor orientation toward a target is a serious challenge. An Inertial Stabilization Platform (ISP) is an effective solution to addressing this challenge. The two-axis yaw–pitch gimbal configuration is a basic method in ISP. Because it is commonly used in many systems, it can be regarded as the archetype for other configurations, such as roll–pitch, mirror stabilization, and four-axis configurations. In this paper, the modeling and control of the two-axis yaw–pitch gimbal are studied.

Many scholars prefer the Newton–Euler method and the Lagrange method to model the two-axis yaw–pitch gimbal. In [3,4,5], the dynamic model of a two-axis yaw–pitch gimbal is established using the Newton–Euler equations. However, when constructing the Newton–Euler equations for rigid bodies, it is necessary to consider the hinge forces between them. This not only increases the number of unknown variables but also complicates the equations.

In [6,7,8], the dynamic model of a two-axis yaw–pitch gimbal is constructed using the Lagrange equations. The Lagrange equations require the calculation of the system’s kinetic and potential energies, followed by solving second-order differential equations to obtain the final dynamic model. The Lagrange equations effectively avoid the analysis of hinge constraint forces between rigid bodies. However, due to the need to solve second-order differential equations, computational efficiency is relatively low.

In order to address the aforementioned issues, this paper proposes the Kane method to establish the dynamic model of the two-axis yaw–pitch gimbal. The Kane method eliminates the disadvantages of methods of classical mechanics (Newton–Euler and Lagrange). The Kane method has the advantage of simplifying the modeling process and increasing computational efficiency. The Kane method has been applied to the dynamic modeling problems of the shipborne Stewart platform [9], space exploration vehicle [10], helicopter hoisting system [11], and manipulator [12].

The object tracking stability of the two-axis gimbal is crucial, as it directly affects the accuracy of measurements from sensors mounted on the gimbal. To ensure the object tracking stability of the two-axis gimbal, it is essential to have an excellent control system.

The PID controller holds a dominant position in various engineering applications, including aerospace, power electronics, and machining. PID controller provides following advantage: simple structure, not relying solely on mathematical models, and being easy to implement in engineering. In order to meet the requirements of high precision, high adaptability, and stable platform control, ref. [13] developed a new fractional-order PID controller. Unlike the classical PID controller, the new controller functions across both differential and integral orders, offering enhanced flexibility in numerical aspects. In order to enhance the performance of the control system, a combination of fuzzy control and PID is utilized and applied to the position tracking of the seeker inertial stabilization platform [14]. In [15], a self-tuning fuzzy PID controller for missile two-axis gimbal is proposed. Additionally, a PID controller with coefficients optimized by using the particle swarm optimization method is designed. Experimental studies reveal that the PID with adjustable coefficients outperforms the PID controller with fixed coefficients. In summary, since it is easy to design and implement, the classical PID controller plays a significant role. Drawing upon the concept of adjustable PID control parameters mentioned in the aforementioned literature, this paper proposes an NPSO-PID controller to address the shortcomings of traditional PSO-PID controllers.

In this paper, a Kane method is proposed for establishing the dynamic model of a two-axis stabilizing gimbal, along with an NPSO-PID control method to ensure object tracking stability by the two-axis stabilizing gimbal. The remainder of this paper is organized as follows: Section 2 establishes dynamic equations of the two-axis yaw–pitch gimbal using Kane method. Section 3 presents control method, and Section 4 conducts simulation verification. Finally, the study is summarized in Section 5.

This paper is dedicated to achieving simplified modeling and excellent object tracking using a two-axis yaw–pitch gimbal system, and its main contributions lie in the following:

(1) A modeling method based on the Kane method is proposed, which avoids the shortcomings of the Newton–Euler method and the Lagrange method in establishing system model.
(2) A NPSO-PID control method is presented to ensure the object tracking stability of the two-axis gimbal. Compared to classical PID controllers, NPSO-PID exhibits superior object tracking capability. Additionally, when compared to traditional PSO-PID controllers, NPSO-PID demonstrates the better optimization of control parameters.

## 2. Kane Equation

The Kane equation [16] is presented as a set of scalar Equation (1)

(1) $$F_{\text{​}}^{\left(r\right)}+F_{\text{​}}^{\left(r\right)*}=0\text{​}\left(r=1,2,3,\ldots ,f\right)$$

where *f* is the number of degrees of freedom of the system. The generalized active forces are given by

(2) $$F_{\text{​}}^{\left(r\right)}=H\cdot V+T\cdot \omega_{\text{​}}^{\left(r\right)}$$

And the generalized inertia forces are given by

(3) $$F_{\text{​}}^{\left(r\right)*}=-H^{*}\cdot V+T^{*}\cdot \omega_{\text{​}}^{\left(r\right)}$$

*H* is the principal vector, and *T* is the principal moment; The vector quantities $\omega_{i}$ and $V_{i}$ are the partial angular velocity and partial velocity, respectively.

### 2.1. Generalized Coordinate and Partial Velocity

**Figure 1:**
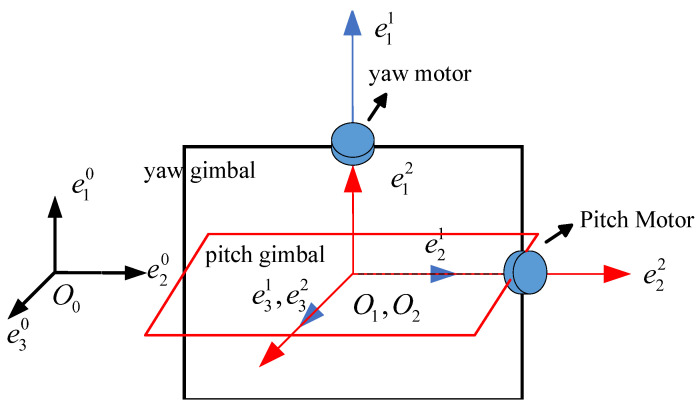
Schematic diagram of two-axis stable gimba.

Consider a two-axis, yaw–pitch stable gimbal system as depicted in Figure 1. In the figure the yaw and pitch gimbal are indicated. The sensor is placed on the pitch gimbal. The gimbals are regarded as rigid bodies.

**Figure 2:**
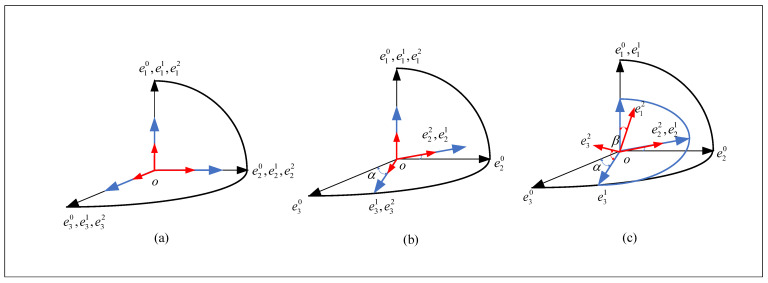
Coordinate system relationship. (**a**) The relationship of the three vector bases at the initial moment. (**b**) The relationship of the three vector bases with the yaw angle. (**c**) The relationship of the three vector bases with both the yaw angle and the pitch angle.

The three orthogonal unit vectors intersecting at point O are denoted as $e_{1},e_{2}$ and $e_{3}$, respectively, and are referred to as the base vectors. They form an orthogonal coordinate system called the basis, denoted as $\left(0,e\right)$, where the symbol for the basis is *e*. $\left(O_{0},e^{0}\right)$ represents the fixed reference base. $\left(O_{1},e^{1}\right)$ is the yaw base vector. $\left(O_{2},e^{2}\right)$ is the pitch base vector. The three vector bases will have the relationship as is shown in Figure 2 during the operating state.

α—relative yaw angle between the yaw gimbal and base coordinate systems.

β—relative pitch angle between pitch and yaw gimbal coordinate systems.

The yaw gimbal coordinate system angular velocity is

(4) $$\left[\begin{array}{c} \omega_{11}\\ \omega_{12}\\ \omega_{13} \end{array}\right]=\left[\begin{array}{c} \dot{\alpha }\\ 0\\ 0 \end{array}\right]$$

The coordinate transformation between the yaw and pitch gimbal coordinate system is given by

(5) $$C_{1}^{2}=\left[\begin{array}{ccc} \cos\beta & 0 & -\sin\beta \\ 0 & 1 & 0\\ \sin\beta & 0 & \cos\beta \end{array}\right]$$

The yaw gimbal coordinate system angular velocity is related to the pitch gimbal coordinate system rate vector by

(6) $$\left[\begin{array}{c} \omega_{21}\\ \omega_{22}\\ \omega_{23} \end{array}\right]=\left[\begin{array}{ccc} \cos\beta & 0 & -\sin\beta \\ 0 & 1 & 0\\ \sin\beta & 0 & \cos\beta \end{array}\right]\left[\begin{array}{c} \dot{\alpha }\\ 0\\ 0 \end{array}\right]+\left[\begin{array}{c} 0\\ \dot{\beta }\\ 0 \end{array}\right]=\left[\begin{array}{c} \dot{\alpha }\cos\beta \\ \dot{\beta }\\ \dot{\alpha }\sin\beta \end{array}\right]$$

${\left[\begin{array}{ccc} \dot{\alpha } & 0 & 0 \end{array}\right]}^{T}$ represents the inertial velocity of the yaw gimbal.

${\left[\begin{array}{ccc} 0 & \dot{\beta } & 0 \end{array}\right]}^{T}$ represents the inertial velocity of the pitch gimbal.

From Figure 2, we can derive the relative generalized velocity ($u_{r}$) of the two-axis stable gimbal, along with its corresponding partial velocities ($v_{1},v_{2}$) and partial angular velocities ($\omega_{1},\omega_{2}$), as summarized in Table 1.

When selecting the generalized velocity, the principle should be to make the partial velocity and partial angular velocity as simple as possible. Therefore, the generalized velocity of the two-axis, yaw–pitch stable gimbal system is ${\left[u_{1},u_{2},u_{3}\right]}^{T}={\left[\dot{\alpha }\cos\beta ,\dot{\beta },\dot{\alpha }\sin\beta \right]}^{T}$.

The generalized velocity is essentially a scalar. The role of partial velocity is to give direction to the generalized velocity. The generalized velocity can be considered as the projection of the true velocity onto the partial velocity. Form Figure 2c the partial angular velocity of the pitch gimbal is represented by ${\left[\omega_{2}^{1},\omega_{2}^{2},\omega_{2}^{3}\right]}^{T}={\left[e_{1}^{2},e_{2}^{2},e_{3}^{2}\right]}^{T}$, and the partial angular velocity of the yaw gimbal is represented by ${\left[\omega_{1}^{1},\omega_{1}^{2},\omega_{1}^{3}\right]}^{T}={\left[\cos\beta e_{1}^{1},0,\sin\beta e_{1}^{1}\right]}^{T}$. Since the system undergoes fixed-axis rotation and the center of mass does not change its position, $v_{1}^{\left(r\right)}=v_{2}^{\left(r\right)}=0\text{​}\left(r=1,2,3\right)$.

The center of mass velocity and angular velocity of each rigid body are shown in Equation (7).

(7) $$\left\{\begin{array}{c} v_{1}=0\\ v_{2}=0\\ \omega_{1}=u_{1}\cos\beta e_{1}^{1}+u_{3}\sin\beta e_{1}^{1}\\ \omega_{2}=u_{1}e_{1}^{2}+u_{2}e_{2}^{2}+u_{3}e_{3}^{2} \end{array}\right.$$

The center of mass acceleration and angular acceleration of each rigid body are calculated as follows by taking the derivative of Equation (7) with respect to time, as in Equation (8)

(8) $$\left\{\begin{array}{c} {\dot{v}}_{1}=0\\ {\dot{v}}_{2}=0\\ {\dot{\omega }}_{1}=\left({\dot{u}}_{1}\cos\beta -u_{1}\dot{\beta }\sin\beta +{\dot{u}}_{3}\sin\beta +u_{3}\dot{\beta }\cos\beta \right)e_{1}^{1}\\ {\dot{\omega }}_{2}={\dot{u}}_{1}e_{1}^{2}+{\dot{u}}_{2}e_{2}^{2}+{\dot{u}}_{3}e_{3}^{2} \end{array}\right.$$

### 2.2. Dynamic Equations

After analyzing the system structure on the two-axis stabilized gimbal, it is clear that the motors’ output forces are the system active forces, denoted as $M_{1}e_{1}^{1}$ and $M_{2}e_{2}^{2}$. $F_{1}$ and $F_{2}$ are defined as the generalized active forces of the two-axis stabilized gimbal, with components in the $u_{r}$ direction denoted as $F_{1}^{\left(r\right)}$ and $F_{2}^{\left(r\right)}$.

The generalized active force in the $u_{1}$ direction is shown in Equation (9).

(9) $$\left.\begin{array}{c} F_{1}^{\left(1\right)}=M_{1}\cos\beta \\ F_{2}^{\left(1\right)}=0 \end{array}\right\}F^{\left(1\right)}=M_{1}\cos\beta$$

The generalized active force in the $u_{2}$ direction is shown in Equation (10).

(10) $$\left.\begin{array}{c} F_{1}^{\left(2\right)}=0\\ F_{2}^{\left(2\right)}=M_{2} \end{array}\right\}F^{\left(2\right)}=M_{2}$$

The generalized active force in the $u_{3}$ direction is shown in Equation (11).

(11) $$\left.\begin{array}{c} F_{1}^{\left(3\right)}=M_{1}\sin\beta \\ F_{2}^{\left(3\right)}=0 \end{array}\right\}F^{\left(1\right)}=M_{1}\sin\beta$$

$F_{1}^{*}$ and $F_{2}^{*}$ are defined as the generalized inertia forces of the two-axis stabilized gimbal, with components in the $u_{r}$ direction denoted as $F_{1}^{\left(r\right)*}$ and $F_{2}^{\left(r\right)*}$.

Existing references [1,17,18] all assume that the rotation axis is the geometric axis of symmetry. Reference [19] experimentally determined that when the gimbal angle varies between $\pm 60^{\circ }$, the product of inertia only varies between $\pm 1\times 10^{-5}\mathrm{kg}\cdot m^{2}$, demonstrating that the impact of the product of inertia on the gimbal rotation is minimal. Therefore, the expression for the moment of inertia used in this paper is as shown in Equation (12).

(12) $$\left\{\begin{array}{c} J^{1}=\mathrm{diag}\left[J_{11},J_{12},J_{13}\right]\\ J^{2}=\mathrm{diag}\left[J_{21},J_{22},J_{23}\right] \end{array}\right.$$

The generalized inertia force in the $u_{1}$ direction is shown in Equation (13).

(13) $$\begin{array}{c} F_{1}^{\left(1\right)*}=-J_{11}\cos\beta \left({\dot{u}}_{1}\cos\beta -u_{1}\dot{\beta }\sin\beta +{\dot{u}}_{3}\sin\beta +u_{3}\dot{\beta }\cos\beta \right)\\ F_{2}^{\left(1\right)*}=u_{2}u_{3}\left(J_{22}-J_{23}\right)-J_{21}{\dot{u}}_{1}\\ F^{\left(1\right)*}=F_{1}^{\left(1\right)*}+F_{2}^{\left(1\right)*}\\ =-J_{11}\cos\beta \left({\dot{u}}_{1}\cos\beta -u_{1}\dot{\beta }\sin\beta +{\dot{u}}_{3}\sin\beta +u_{3}\dot{\beta }\cos\beta \right)\\ +u_{2}u_{3}\left(J_{22}-J_{23}\right)-J_{21}{\dot{u}}_{1} \end{array}$$

The generalized inertia force in the $u_{2}$ direction is shown in Equation (14).

(14) $$\begin{array}{c} F_{1}^{\left(2\right)*}=0\\ F_{2}^{\left(2\right)*}=u_{1}u_{3}\left(J_{23}-J_{21}\right)-J_{22}{\dot{u}}_{2}\\ F^{\left(2\right)}=F_{1}^{\left(2\right)*}+F_{2}^{\left(2\right)*}\\ =u_{1}u_{3}\left(J_{23}-J_{21}\right)-J_{22}{\dot{u}}_{2} \end{array}$$

The generalized inertia force in the $u_{3}$ direction is shown in Equation (15).

(15) $$\begin{array}{c} F_{1}^{\left(3\right)*}=-J_{11}\sin\beta \left({\dot{u}}_{1}\cos\beta -u_{1}\dot{\beta }\sin\beta +{\dot{u}}_{3}\sin\beta +u_{3}\dot{\beta }\cos\beta \right)\\ F_{2}^{\left(3\right)*}=-u_{1}u_{2}\left(J_{22}-J_{21}\right)-J_{23}{\dot{u}}_{3}\\ F^{\left(3\right)}=F_{1}^{\left(3\right)*}+F_{2}^{\left(3\right)*}\\ =-J_{11}\sin\beta \left({\dot{u}}_{1}\cos\beta -u_{1}\dot{\beta }\sin\beta +{\dot{u}}_{3}\sin\beta +u_{3}\dot{\beta }\cos\beta \right)-u_{1}u_{2}\left(J_{22}-J_{21}\right)-J_{23}{\dot{u}}_{3} \end{array}$$

According to the Kane equation, Equation (16) is obtained by utilizing Equation (14).

(16) $$\begin{array}{c} M_{2}=F^{\left(2\right)}=-F^{\left(2\right)*}\\ =J_{22}\ddot{\beta }-\frac{1}{2}{\dot{\alpha }}^{2}\sin2\beta \left(J_{23}-J_{21}\right) \end{array}$$

Equation (17) is obtained by utilizing Equations (13) and (15).

(17) $$\begin{array}{c} M_{1}=\cos\beta F^{\left(1\right)}+\sin\beta F^{\left(3\right)}=-\cos\beta F^{\left(1\right)*}-\sin\beta F^{\left(3\right)*}\\ =\left(J_{11}+J_{21}\cos^{2}\beta +J_{23}\sin^{2}\beta \right)\ddot{\alpha }+\dot{\alpha }\dot{\beta }\left(J_{23}-J_{21}\right)\sin2\beta \end{array}$$

The results of Equations (15) and (16) are in agreement with reference [20], which used the Newton–Euler method to establish the dynamic equations. The paper demonstrates the correctness of the two-axis yaw–pitch gimbal dynamics model established using the Kane method.

To further illustrate the advantages of the Kane method modeling, Table 2 summarizes the number of forces to be considered when modeling two-axis and three-axis stabilized gimbals using the Newton–Euler method and Kane method.

When modeling a two-axis stabilized gimbal using the Newton–Euler method, in addition to considering the driving force of each gimbal’s motor, the imbalance force of its own frame, and the rotational inertia force, there are also forces to account for the imbalance between the two gimbals and the rotational coupling force between them, totaling eight forces. Compared to the Kane method, it requires the consideration of two additional forces.

When modeling a three-axis stabilized gimbal using the Kane method, it only requires defining an additional generalized coordinate for the roll motion on top of the two-axis stabilized gimbal model. The number of forces considered increases from 6 to 12.

The three-axis stabilized gimbal consists of inner, middle, and outer gimbals, each responsible for pitch, yaw, and roll motion, respectively. When using the Newton–Euler method for modeling, it requires analyzing 16 forces. Each gimbal is subjected to the driving force of the motor, the imbalance force of its own gimbal, and the rotational inertia force, totaling nine forces for the three gimbals. The remaining seven forces represent the interactions between the gimbals, including: the imbalance force of the inner gimbal acting on the middle gimbal, the rotational coupling force of the inner gimbal on the middle gimbal, the coupling force between the middle and outer gimbal, the coupling force between the inner and outer gimbal, the imbalance force of the inner gimbal acting on the outer gimbal, the imbalance force of the middle gimbal acting on the outer gimbal, and the combined imbalance force of the inner and middle gimbal acting on the outer gimbal. Compared to the Kane method, it requires the consideration of four additional forces.

The advantages of the Kane method modeling become more apparent as the number of rigid bodies in the system increases. The Kane method provides a convenient way of deriving the dynamic equations of motion for complex multibody systems that have several degrees of freedom [21].

### 2.3. Motor Torque Equation

Due to the fact that stabilizing platforms generally employ DC torque motors as active motors, the motor armature voltage is given as follows:

(18) $$U=L\frac{di}{dt}+\mathrm{Ri}+E_{g}$$

where U is the voltage across the motor armature, *L* is the motor armature inductance, *i* is the motor armature current, R is the motor armature resistance, and $E_{g}$ is the motor back electromotive force. When the motor operates in a steady state, the voltage across the inductance terminals is zero within a cycle, so the motor armature voltage equation can be simplified to

(19) $$U=\mathrm{Ri}+E_{g}$$

For torque motors

(20) $$\left\{\begin{array}{c} E_{g}=K_{e}n\\ M=iK_{T}\\ n=9.55\omega \end{array}\right.$$

where $K_{e}$ is the motor back electromotive force coefficient, *n* is the motor speed, $K_{T}$ is the motor torque coefficient, ω is the motor angular velocity. In practical motor systems, the adoption of current negative feedback ensures stable currents. This, in turn, establishes linear relationships between the control voltage and armature current, as well as between the control voltage and output torque. The relationship between the control voltage and the motor output torque is shown in the formula below, where K is the proportional coefficient, T is the motor output torque, and U is the motor control voltage.

(21) T=KU

### 2.4. Disturbance Factors of the Stabilized Gimbal

In electromechanical control systems, friction torque can significantly affect the control accuracy of the motor. Therefore, this section conducts a modeling analysis of the friction torque. A Stribeck [22] friction torque model is established as shown in Equation (22).

(22) T2fvp=f2c+f2s−f2ce−v2v2v2sv2s2+f2vv2

In (21), $T_{2f}\left(t\right)$ is the friction torque acting on the pitch gimbal, $f_{2c}$ is the maximum static friction of the pitch gimbal, $f_{2s}$ is the Coulomb friction of the pitch gimbal, $v_{2s}$ is the critical velocity for boundary friction of the pitch gimbal, and $f_{2v}$ is the viscous friction coefficient of the pitch gimbal. Therefore, the torque applied to the pitch gimbal is the vector sum of the motor driving torque, the friction torque of the pitch gimbal, and other external disturbance torques acting on the pitch gimbal, i.e.

(23) $$M_{2}=T_{2}\left(t\right)-T_{2w}\left(t\right)-T_{2f}\left(v\right)$$

$T_{2}\left(t\right)$ is the driving torque of the pitch gimbal motor, $T_{2w}\left(t\right)$ is the sum of other disturbance torques acting on the pitch gimbal, and $T_{2f}\left(v\right)$ is the friction torque of the pitch gimbal. Define $T_{2d}$ as the resultant disturbance torque of the pitch gimbal.

(24) $$T_{2d}=T_{2f}+T_{2w}$$

$T_{2f}$ and $T_{2w}$ are the friction torque and other disturbance torques of the pitch gimbal, respectively.

Similarly, the resultant torque of the yaw gimbal is:

(25) $$M_{1}=T_{1}\left(t\right)-T_{1w}\left(t\right)-T_{1f}\left(v\right)$$

$T_{1}\left(t\right)$ is the driving torque of the yaw gimbal motor, $T_{1w}\left(t\right)$ is the sum of other disturbance torques acting on the yaw gimbal, and $T_{1f}\left(v\right)$ is the friction torque of the yaw gimbal. Define $T_{1d}$ as the resultant disturbance torque of the yaw gimbal.

(26) $$T_{1d}=T_{1f}+T_{1w}$$

$T_{1f}$ and $T_{1w}$ are the friction torque and other disturbance torques of the yaw gimbal, respectively.

### 2.5. Lyapunov Proof

Taking the pitch gimbal as an example, construct the pitch gimbal Lyapunov function

(27) $$V=\frac{1}{2}\dot{\beta }$$

Then:

(28) $$\dot{V}=\dot{\beta }\ddot{\beta }$$

(29) $$\ddot{\beta }=\frac{M_{2}+\frac{1}{2}{\dot{\alpha }}^{2}\sin2\beta \left(J_{23}-J_{21}\right)}{J_{22}}$$

(30) $$\dot{V}=\dot{\beta }\ddot{\beta }=\dot{\beta }\frac{M_{2}+\frac{1}{2}{\dot{\alpha }}^{2}\sin2\beta \left(J_{23}-J_{21}\right)}{J_{22}}$$

(1) When β undergoes small angle changes, and $\dot{\beta }\Rightarrow 0$, $\dot{V}=0$, i.e. the Lyapunov origin is stable.

(2) When $J_{23}=J_{21}$, then $\dot{V}=\frac{M_{2}\dot{\beta }}{J_{22}}$, where $J_{22}$ is a constant. When $\dot{\beta }$ and $M_{2}$ have opposite signs, $\dot{V}<0$, the pitch gimbal is stable.

Except for the above two special cases, to ensure the stability of the platform, only $M_{2}+\frac{1}{2}{\dot{\alpha }}^{2}\sin2\beta \left(J_{23}-J_{21}\right)\equiv 0$ then $\dot{V}=0$, which results in Lyapunov stability at the origin. Similarly, the stability of the yaw gimbal can also be proven. It is challenging for torque motors alone to meet the requirements of the above equations in the system. Therefore, control system design is necessary to ensure stability.

## 3. Controller Design

According to analysis in Section 2, it can be observed that there exists a complex nonlinear relationship between the deflection angles of the two-axis gimbal and the torque motors. To adapt this nonlinear relationship, it is necessary to use controllers in the two-axis stable gimbal system.

**Figure 3:**
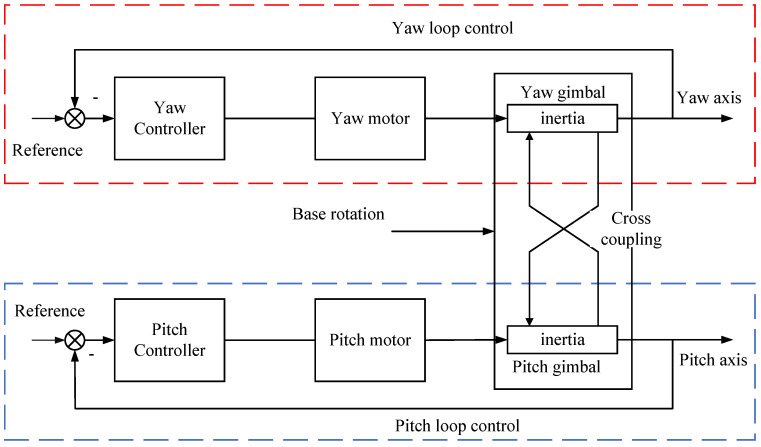
Two-axis stable gimbal control system.

The two-axis gimbal control system comprises two loops: the yaw-axis control loop and the pitch-axis control loop. The system control diagram is shown in Figure 3. To ensure the stability of the gimbal, an NPSO-PID controller is designed for the yaw/pitch controller module.

Particle Swarm Optimization (PSO) is a population-based metaheuristic algorithm inspired by the foraging behavior of birds. Its primary objective is to discover the maximum food source (global optimal solution) within a given search space. Each particle in the population represents a potential solution, and they collaborate by exchanging information to converge towards the best solution [23].

The entire optimization process of the particle swarm algorithm proceeds as follows: Firstly, the initial positions and velocities of particles in the swarm are initialized. Then, the iteration process of the particle swarm algorithm is carried out. During each iteration, the fitness value of each particle is computed. Based on these fitness values, the particles update their velocity and position information until the iteration process concludes. After the iteration, the global optimal particle is obtained, and the parameter of the controller also reaches its optimal state under the present conditions, thereby enhancing the controller’s control capability.

In each iteration of the algorithm, both the position and velocity of particles are updated simultaneously. The particle’s position represents the solution being sought, while the velocity determines the adjustment step size for each iteration. To avoid blind searching by particles in the algorithm, it is customary to set upper and lower bounds for the position and step size based on the specific range of the actual control quantity. The update method for particle position and step size is as follows:

(31) $$\left\{\begin{array}{c} v_{id}^{k+1}=w\times v_{id}^{k}+c_{1}\times r_{1}\times \left(p_{id}-x_{id}^{k}\right)+c_{2}\times r_{2}\times \left(p_{gd}-x_{id}^{k}\right)\\ x_{id}^{k+1}=x_{id}^{k}+v_{id}^{k+1} \end{array}\right.$$

(32) $$w=w_{\max}-\left(w_{\max}-w_{\min}\right)*\frac{k}{k_{sum}}$$

In (31), $v_{id}^{k+1}$ denotes the velocity information of the *i*-th particle in the *d*-th dimension at the $\left(k+1\right)$-th iteration, while $x_{id}^{k+1}$ represents the position information of the *i*-th particle in the *d*-th dimension at the $\left(k+1\right)$-th iteration. The variables *c*1 and *c*2 are learning factors, and *w* stands for the inertia weight. $p_{gd}$ represents the global best value, while $p_{id}$ denotes the historical best value of individual particles. The *k*-th iteration corresponds to the time $t=t_{k}$, and the $\left(k+1\right)$-th iteration corresponds to the time $t=t_{k+1}$. Therefore, the terms $v_{id}^{k+1}$, $x_{id}^{k+1}$, $v_{id}^{k}$, and $x_{id}^{k}$ in Equation (31) all contain time information.

In (32), $w_{\max}$ represents the maximum weight, $w_{\min}$ represents the minimum weight. *k* denotes the current iteration number, and $k_{sum}$ represents the total number of iterations.

In PSO algorithms, to evaluate the quality of the sought values, a fitness function is introduced. Among all the fitness functions considered thus far, the most comprehensive and reasonable evaluation criterion is the ITAE method [24].

(33) $$J=\int_{0}^{\infty }t\left|e\left(t\right)\right|dt$$

The general form of a PID [25] controller is as follows:

(34) $$u\left(t\right)=K_{p}e\left(t\right)+K_{i}\int_{0}^{t}e\left(\tau \right)d\tau +K_{d}\frac{de\left(t\right)}{dt}$$

**Figure 4:**
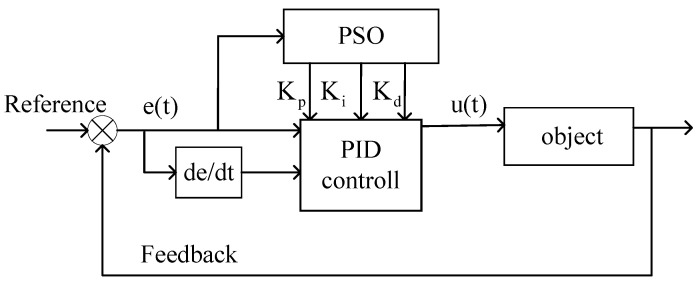
PSO-PID control system.

However, in practical engineering, it is impossible to directly handle the differential terms of a continuous-time PID algorithm. A commonly used method is to discretize the continuous-time PID controller, replacing integration with summation and differentiation with finite differences. After discretizing the proportional, integral, and derivative terms, the form of the PID controller is as follows:

(35) $$u\left(k\right)=K_{p}e\left(k\right)+K_{i}\sum_{i=1}^{k}e\left(i\right)\Delta t+K_{d}\frac{e\left(k\right)-e\left(k-1\right)}{\Delta t}$$

where $u\left(k\right)$ represents the controller output signal at time *k*, $e\left(k\right)$ denotes the system error at time *k*, and Kp,​Ki and Kd are the weighted values of the system error signal and its integral and derivative components, respectively. PSO-PID utilizes the optimization characteristics of the PSO algorithm to adjust the three parameters of the PID controller, thereby achieving stability in the control system. The control diagram is depicted in Figure 4. The pseudocode for the PSO-PID model is presented in Table 3.

**Figure 5:**
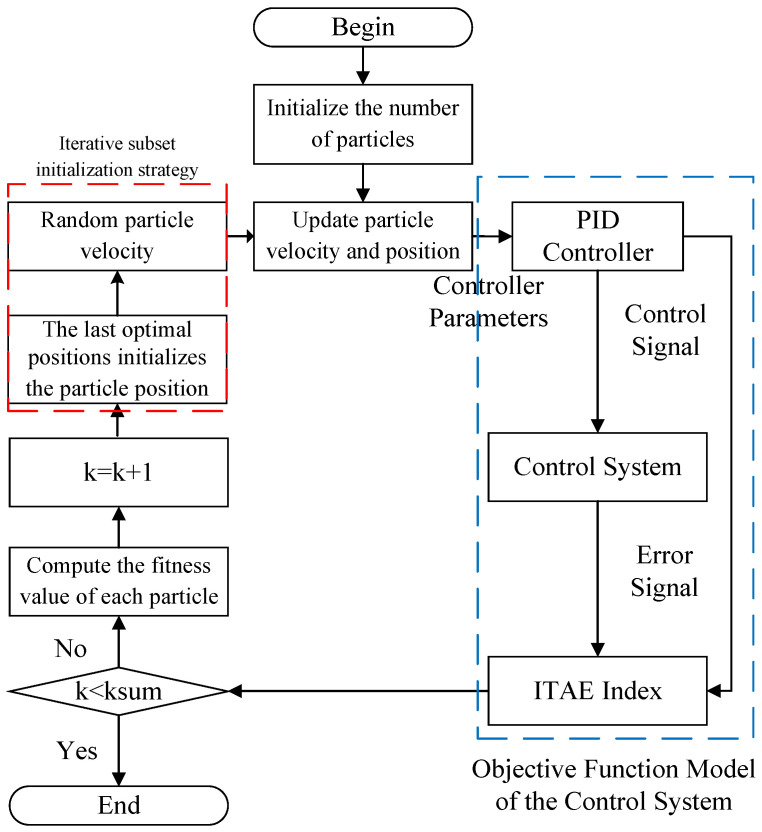
NPSO-PID flowchart.

The traditional PSO-PID algorithm is presented Table 3. As can be seen, the traditional PSO-PID algorithm does not fully utilize all particles during the process of finding the optimal position. Specifically, particle initialization follows a random distribution. So when one particle is determined as optimal (denoted as A1), the other particles are defined as A2,A3,…,AN based on their fitness values from smallest to largest. During the iteration process, AN need to continually move towards A1 to achieve a smaller fitness value. However, calculating the fitness value of AN after each iteration update may result in an unnecessary computational burden. Because AN may encounter situations where no solution is found, the computational burden for this is extremely significant. To address this issue, we propose an iterative subset initialization strategy. Before updating velocity and position in each iteration, all particles are initialized at the position of the previous global optimum fitness value, followed by assigning them random initial velocities to position them near the previous global optimum position. This process is repeated until the iteration ends. This modified PSO algorithm is named NPSO. The pseudocode for the NPSO-PID model is presented in Table 4, and the flowchart is shown in Figure 5.

In Figure 5, it can be clearly seen that compared to the traditional PSO-PID, the NPSO-PID includes an additional red-boxed section, which represents the iterative subset initialization strategy proposed in this paper. The purpose of adding this module is to enhance the optimization performance of PSO, thereby providing the NPSO-PID with better control performance.

## 4. Simulink Results and Analysis

This section takes the two-axis stable gimbal as the research subject to analyze the stability of object tracking of the two-axis gimbal. The initial parameter settings of the two-axis stable gimbal are shown in Table 5.

### 4.1. Comparison of NPSO-PID and PID

According to Section 3, an NPSO-PID controller was designed and compared with the classical PID controller in terms of control performance. From Equations (16) and (17), it is evident that the pitch gimbal stabilizes, the yaw gimbal can also stabilize rapidly, and vice versa. Therefore, to achieve faster response speed and better stability, yaw controllers and pitch controllers adopt the same set of control parameters. The parameters of the classical PID controller (Kp, Ki, Kd are 4.08, 0.8703, and 7.002, respectively) are obtained from reference [26]. Control parameters of NPSO-PID are obtained after optimization by NPSO. The initial parameter settings of NPSO are shown in Table 6.

**Figure 6:**
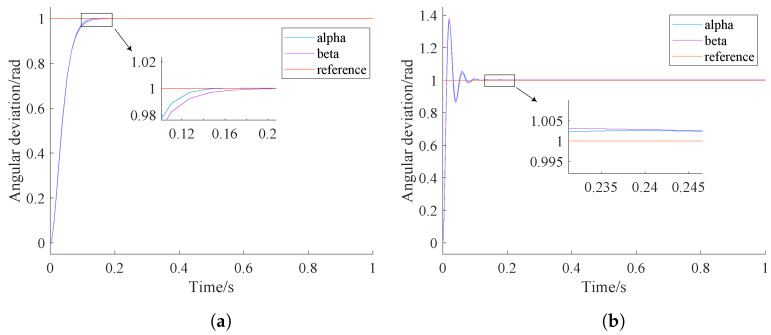
Controller step tracking comparison chart. (**a**) NPSO-PID tracking. (**b**) PID tracking.

**Figure 7:**
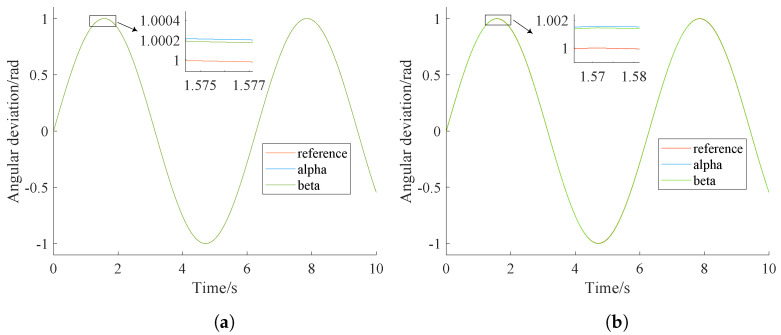
Controller sinusoidal tracking effect comparison. (**a**) NPSO-PID tracking. (**b**) PID tracking.

The control parameters optimized by NPSO for step signal tracking and sinusoidal signal tracking are [46.348, 0, 1.75] and [46.754, 0, 49.098], respectively. The simulation results of NPSO-PID and classical PID are shown in Figure 6 and Figure 7. These results are further summarized in Table 7.

In Figure 6, it can be clearly seen that both the NPSO-PID and the classical PID can effectively track step signals, but the classical PID has a 39% overshoot, and has failed to meet the tracking accuracy requirement in which tracking error should not exceed $0.5^{\circ }$ [27].

Figure 7 indicates that both controllers show good control performance in tracking sinusoidal signals. However, compared with the classical PID controller, the tracking accuracy of the NPSO-PID controller improves by an order of magnitude.

From Figure 6 and Figure 7, it can be observed that both controllers achieve a faster response speed and better stability with the same control parameters.

From the analysis of Table 7, it can be seen that when the NPSO-PID controller tracks a step signal, the yaw gimbal and pitch gimbal stabilize at 0.16 s and 0.2 s, respectively, with a tracking error of 0. When the PID controller tracks a step signal, both the yaw gimbal and pitch gimbal stabilize at 0.24 s, but there is a tracking error of 0.003 rad.

When the NPSO-PID controller tracks a sine signal, the tracking errors for the yaw gimbal and pitch gimbal are 0.00026 rad and 0.00028 rad, respectively. In contrast, when the PID controller tracks a sine signal, both the yaw gimbal and pitch gimbal have a tracking error of 0.001 rad, which is an order of magnitude higher than that of the NPSO-PID controller.

This demonstrates that the NPSO-PID controller has better control performance.

### 4.2. NPSO-PID Optimization Accuracy Verification

**Figure 8:**
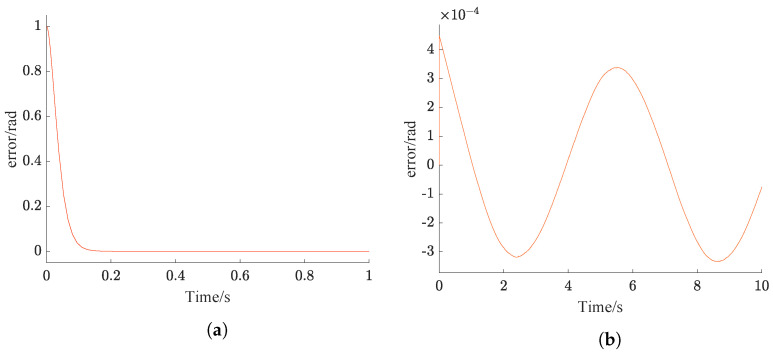
Accuracy verification. (**a**) Step error. (**b**) Sinusoidal error.

The difference between the desired value and the system feedback is referred to as error, which effectively reflects the overall performance of the designed controller. The error variation of the NPSO-PID controller is illustrated in Figure 8.

From Figure 8a, the tracking error of NPSO-PID for the step signal rapidly decreases, and the error is essentially eliminated within 0.2 s. From Figure 8b, it can be seen that the tracking error of NPSO-PID for the sine signal exhibits a noticeable initial increase, ultimately stabilizes and keeps within the range of $\pm 3\times 10^{-4}\;\mathrm{rad}$.

### 4.3. Comparison between NPSO-PID and PSO-PID

**Figure 9:**
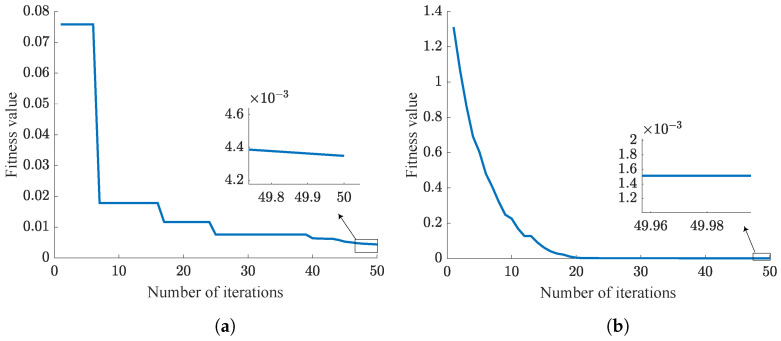
Case 1 results. (**a**) PSO-PID. (**b**) NPSO-PID.

**Figure 10:**
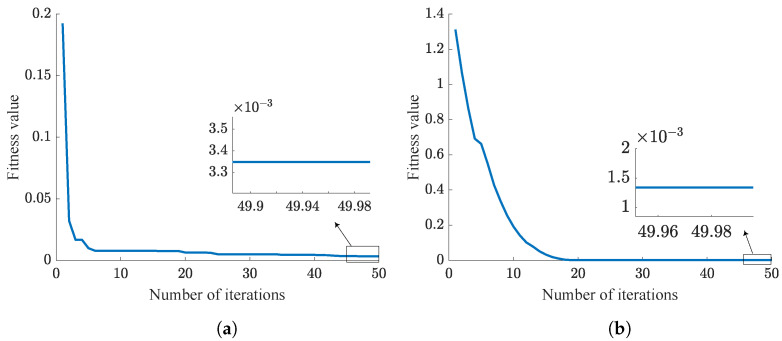
Case 2 results. (**a**) PSO-PID. (**b**) NPSO-PID.

**Figure 11:**
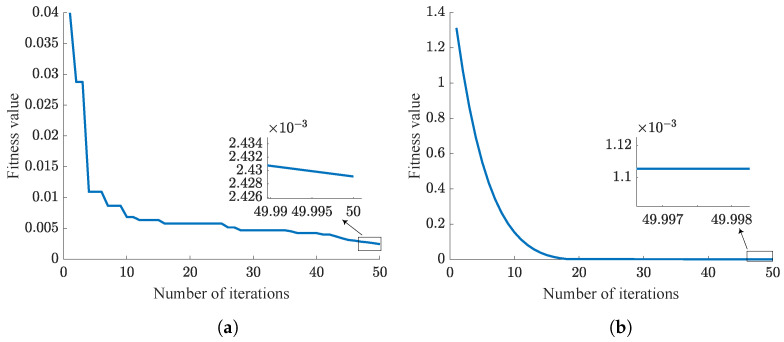
Case 3 results. (**a**) PSO-PID. (**b**) NPSO-PID.

**Figure 12:**
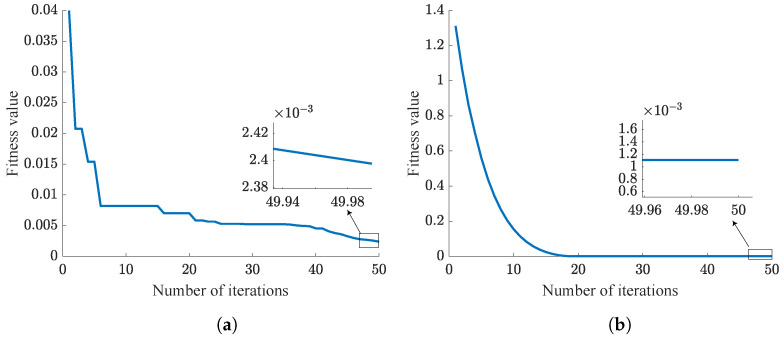
Case 4 results. (**a**) PSO-PID. (**b**) NPSO-PID.

**Figure 13:**
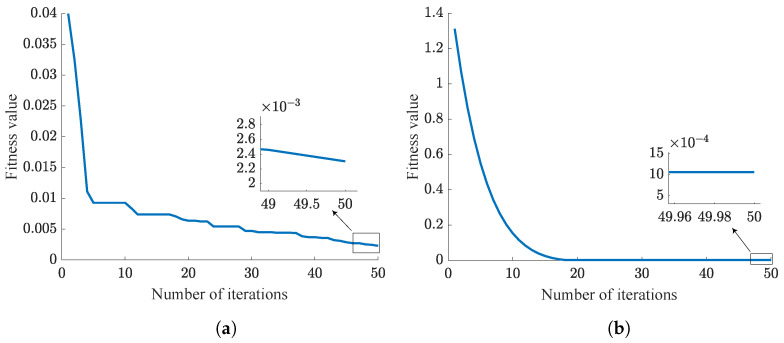
Case 5 results. (**a**) PSO-PID. (**b**) NPSO-PID.

This section mainly compares the performance of the traditional PSO-PID algorithm with the NPSO-PID algorithm. From Equation (33), it is evident that smaller fitness value indicates better control parameter. As is shown in Table 8, five challenging experiments are designed, each aimed at tracking step signals. Simulation results are illustrated from Figure 9, Figure 10, Figure 11, Figure 12 and Figure 13. These results are further summarized in Table 9.

In the PSO algorithm, the fewer the particles, the more difficult it is to find the optimal control parameters. To test and compare the sensitivity of the PSO-PID and NPSO-PID optimization algorithms to the number of particles, we designed five sets of experiments as shown in Table 8. These five sets of experiments have the same number of iterations, but the number of particles gradually increases.

According to the trend of the curves, it can be concluded that NPSO-PID demonstrates better robustness compared to the PSO-PID algorithm. This conclusion is consistent with the inference in Section 3, where all particles are randomly distributed near the last iteration’s best position before each iteration begins, fully utilizing each particle search, thereby achieving better fitness values. However, NPSO-PID exhibits a higher initial fitness value. This is attributed to the strategy of initial subset selection in iteration, where velocity assignment occurs. When particles inherit the last optimal positions, random velocities are assigned to particles. This process disperses particles around the last optimal positions, resulting in higher initial fitness values.

Analysis of Table 9 reveals that although NPSO-PID takes slightly longer for optimization compared to PSO-PID, it delivers superior optimization results. This difference is clearly observed in Case 1, where the fitness value of NPSO-PID is only 1/3 of that of PSO-PID. Summarizing the five cases, it can be concluded that NPSO-PID exhibits better optimization control parameter capabilities.

### 4.4. The Relationship between Particle Number and Fitness Value

**Figure 14:**
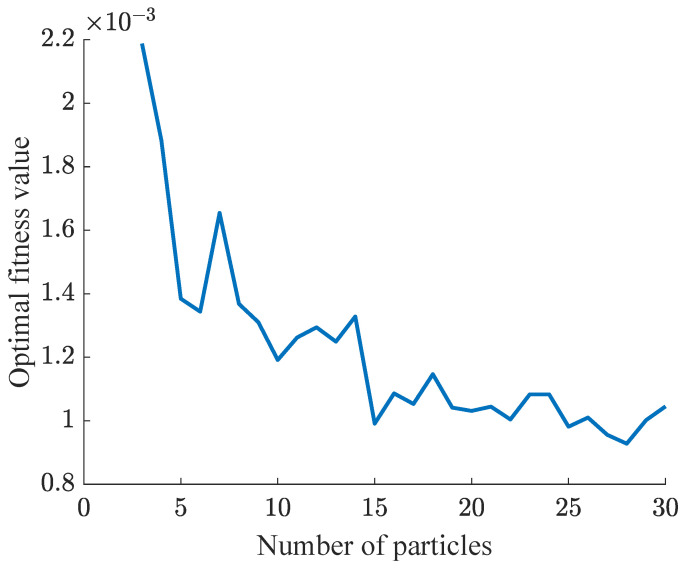
Relationship between particle count and fitness.

In this section, the relationship between different particle counts and optimal fitness in NPSO-PID is investigated. The initial number of particles is set to 3, incremented by 1 each time, and the study concludes when there are 30 particles. Each particle count undergoes 50 iterations. The simulation results are depicted in Figure 14.

From Figure 14, it is evident that the overall trend shows an improvement in the optimal fitness with an increase in the number of particles. As the number of particles increases, the range for finding the optimal fitness also expands, resulting in an improvement in the optimal fitness.

However, in certain cases, the optimal fitness value may increase. This is primarily due to two reasons:

(1) The initialization distribution of particles is random. Some particles are fortunately initialized near the optimal fitness value, while others are unfortunately initialized far from the optimal fitness value, and may not find it by the end of the iterations.
(2) The particle velocity is random, causing the particle movement direction to be random. Increasing the number of iterations can mitigate these deviations.

### 4.5. Analysis of the Influence of Friction

**Figure 15:**
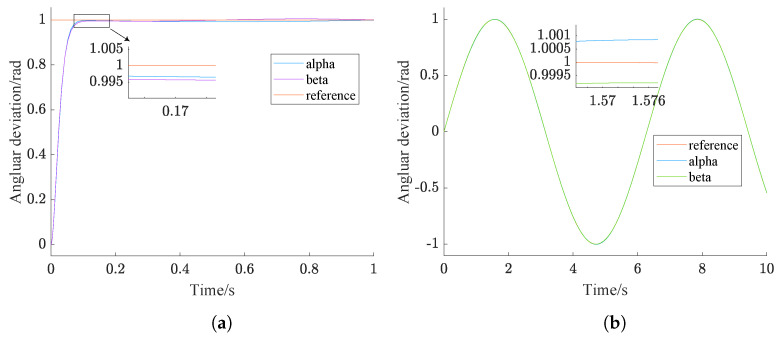
Controller tracking effect. (**a**) NPSO —PID step tracking. (**b**) NPSO —PID sinusoidal tracking.

According to the literature [19], the disturbance torque applied to the pitch gimbal is $T_{1d}=0.1\sin\left(2\pi \cdot t\right)\left(N\times m\right)$, and the disturbance torque applied to the yaw gimbal is $T_{2d}=0.2\sin\left(\pi \cdot t\right)\left(N\times m\right)$. The control parameters optimized by NPSO for step signal tracking and sinusoidal signal tracking are [100, 0.249, 2.581] and [99.973, 0, 99.973], respectively. The simulation results are shown in Figure 15. A comparison of the tracking results for step and sine signals with no friction disturbance torque is presented in Table 10.

From Figure 15, it can be seen that after adding disturbance torque to the system, the NPSO-PID exhibits a steady-state tracking error of 0.005 rad when tracking a step signal. The tracking error range for the NPSO-PID when tracking a sine signal is within ±0.008 rad.

From Table 10, it can be seen that although the control performance of the NPSO-PID decreases after adding disturbance torque, the tracking error remains within the allowable range. This indicates that the NPSO-PID has excellent control performance.

## 5. Conclusions

This paper establishes dynamic model of a two-axis stable gimbal based on the Kane method. Its purpose is to simplify the analysis process during multi-rigid body modeling to facilitate the improvement of computing efficiency and the need for real-time control. To address the issue of the object tracking stability of the two-axis gimbal, an NPSO-PID control method is proposed to meet real-time task requirements.

The dynamic modeling of the two-axis gimbal using the Kane method overcomes the drawbacks of traditional methods (Newton–Euler and Lagrange). Specifically, it avoids considering the hinge constraint forces between rigid bodies in the Newton–Euler method and eliminates the need to calculate second-order derivatives in Lagrange. The NPSO-PID controller designed in this paper has the advantages of higher tracking accuracy and smaller overshoot compared to the classical PID controller. In comparison with the traditional PSO-PID, NPSO-PID demonstrates better optimization of control parameters. However, the NPSO-PID still faces issues of a relatively large initial fitness value and a slight decrease in control performance after the addition of disturbance torque. These are directions for future research.

## Figures and Tables

**Table 1:** Generalized velocity ($u_{r}$), partial velocity ($v_{1},v_{2}$), and partial angular velocity ($\omega_{1},\omega_{2}$).

r	1	2	3
$u_{r}$	$\dot{\alpha }\cos\beta$	$\dot{\beta }$	$\dot{\alpha }\sin\beta$
$\omega_{1}^{\left(r\right)}$	$\cos\beta e_{1}^{1}$	0	$\sin\beta e_{1}^{1}$
$\omega_{2}^{\left(r\right)}$	$e_{1}^{2}$	$e_{2}^{2}$	$e_{3}^{2}$
$v_{1}^{\left(r\right)}$	0	0	0
$v_{2}^{\left(r\right)}$	0	0	0

**Table 2:** The number of forces considered in the gimbal modeling process using the Newton–Euler method and the Kane method.

Gimbal Structure	Newton–Euler	Kane Method
two-axis gimbal	8	6
three-axis gimbal	16	12

**Table 3:** PSO-PID algorithm flow.

PSO-PID Calculation Process
**Input:** The number of particles, the values of $k_{sum}$, $w_{\max}$, $w_{\min}$, and the ranges of Kp,​Ki and Kd
**Output:** The optimal control parameters.
1. Initialize the positions and velocities of the particles.
2. Compute the fitness value of each particle based on Equations (33) and (34), and
determine $p_{gd}$ and $p_{id}$.
3. While ( $k<k_{sum}$ ) do
4. Update particle velocity and position according to Equations (25) and (26).
5. Compute the fitness value of each particle based on Equations (33) and (34), and
determine $p_{gd}$ and $p_{id}$.
6. k+1
7. End while

**Table 4:** NPSO-PID algorithm flow.

NPSO-PID Calculation Process
**Input:** The number of particles, the values of $k_{sum}$, $w_{\max}$, $w_{\min}$, and the ranges of Kp,​Ki and Kd
**Output:** The optimal control parameters.
1. Initialize the positions and velocities of the particles.
2. Compute the fitness value of each particle based on Equations (33) and (34), and
determine $p_{gd}$ and $p_{id}$.
3. While ( $k<k_{sum}$ ) do
4. The last $p_{gd}$ initializes the particle position
5. Random particle velocity
6. Update particle velocity and position according to Equations (25) and (26).
7. Compute the fitness value of each particle based on Equations (33) and (34), and
determine $p_{gd}$ and $p_{id}$.
8. k+1
9. End while

**Table 5:** Two-axis stable gimbal initialization parameters.

Parameter	Value
Rotor inertia J11	0.005954 $\mathrm{kg}\times m^{2}$
Rotor inertia J21	0.001102 $\mathrm{kg}\times m^{2}$
Rotor inertia J22	0.004682 $\mathrm{kg}\times m^{2}$
Rotor inertia J23	0.001122 $\mathrm{kg}\times m^{2}$
Torque constant K1	0.2593 N×m/V
Torque constant K2	0.1852 N×m/V
Inertia α,β	0 rad

**Table 6:** NPSO-PID initial parameter configuration.

Parameter	Value
c1,c2	2
Iterations	50
Number of particles	30
Weight coefficient range	[0.4, 2]
Searching for speed	[−1, 1]
PID value range	[0, 50]

**Table 7:** Comparison of parameters between NPSO-PID and traditional PID.

	Yaw Gimbal			Pitch Gimbal	
	Stable Time	Tracking Error		Stable Time	Tracking Error
setp PID	0.24	0.003		0.24	0.003
setp NPSO-PID	0.16	0		0.2	0
sinusoidal PID	-	0.001		-	0.001
sinusoidal NPSO-PID	-	0.00026		-	0.00028

**Table 8:** Experimental setup.

	Particle	Number of Iteration
Case1	5	50
Case2	10	50
Case3	15	50
Case4	20	50
Case5	25	50

**Table 9:** Performance comparison between PSO-PID and NPSO-PID.

	PSO-PID			NPSO-PID	
	Stable Time	Tracking Error		Stable Time	Tracking Error
1	0.004349	13.547s		0.001515	18.204s
2	0.003048	30.387s		0.001337	35.015
3	0.002429	45.359s		0.001106	47.397s
4	0.002397	62.777s		0.001111	74.877s
5	0.002303	92.255s		0.001059	106.918s

**Table 10:** Comparison of the system with and without friction.

Reference Signal	With and Without Friction	Yaw Gimbal		Pitch Gimbal	
		Stable Time	Tracking Error	Stable Time	Tracking Error
step	with	0.12	0.0025	0.12	0.005
	without	0.16	0	0.2	0
sinusoidal	with	-	0.0008	-	0.0008
	without	-	0.00026	-	0.00028

## Data Availability

Data are contained within the article.
